# Deflating inflation: the connection (or lack thereof) between decisional and metacognitive processes and visual phenomenology

**DOI:** 10.1093/nc/niz015

**Published:** 2019-11-15

**Authors:** Greyson Abid

**Affiliations:** Department of Philosophy, University of California, Berkeley, 314 Moses Hall, Berkeley, CA 94720, USA

**Keywords:** metacognition, consciousness, richness of visual experience, subjective inflation

## Abstract

Vision presents us with a richly detailed world. Yet, there is a range of limitations in the processing of visual information, such as poor peripheral resolution and failures to notice things we do not attend. This raises a natural question: How do we seem to see so much when there is considerable evidence indicating otherwise? In an elegant series of studies, Lau and colleagues have offered a novel answer to this long-standing question, proposing that our sense of visual richness is an artifact of decisional and metacognitive deficits. I critically evaluate this proposal and conclude that it rests on questionable presuppositions concerning the relationship between decisional and metacognitive processes, on one hand, and visual phenomenology, on the other.

## Introduction

Contemporary discussions in philosophy, psychology, and vision science have been concerned with the sense of phenomenological richness associated with vision. As an illustration, consider the view from Coit Tower in San Francisco. On a sunny day, you can witness hundreds of buildings, towers, cars, and pedestrians from the top of the tower. Unlike the other sense modalities, vision is particularly well suited to revealing the richly detailed cityscape. From the top of the tower, I can smell little more than the ocean and hear only the bustle of passing cars. My senses of touch and taste are both too proximal in nature to reveal the distal landscape. It is only when I open my eyes that I am able to witness the breathtaking view of the city.

Nevertheless, the vision’s unique capacity to reveal the richly detailed world around us is quite puzzling given the battery of different limitations in the processing of visual information. To start, visual resolution is greatly limited in one’s periphery. This can be appreciated by noting that we cannot identify a playing card presented in the periphery (Dennett 1991) and fail to discern significant peripheral distortions in images (Anstis 1998; Freeman and Simoncelli 2011) [Note that these examples do not demonstrate that color vision is lacking in the periphery (cf. Schwitzgebel 2008; Lau and Rosenthal 2011; Cohen *et al.* 2016). When cortical magnification is controlled for, there are no significant differences between color vision in foveal and peripheral regions of the visual field (see Tyler 2015; Haun *et al.* 2017)]. A related phenomenon is visual crowding, which occurs when a target in the periphery is surrounded by distractors (i.e. “flankers”). Such targets are identifiable if displayed in isolation but are difficult to identify when placed in a crowded display (Whitney and Levi 2011). Finally, phenomena such as change and inattentional blindness show that if we are not paying attention to something, it is unlikely that we will notice it (Simons 2000; Simons and Ambinder 2005; Simons and Rensink 2005). Instead, we will often claim that we do not see it, even if we are staring right at it.

The foregoing remarks raise a puzzle: How do we seem to see so much when there is considerable evidence indicating otherwise? This vision science mystery has been the subject of a great deal of inquiry [O’Regan and Noë (2001; see also Noë 2002) argue that a puzzle only arises if one assumes that the richness associated with visual experience must be situated “in the head.” They suggest that the rich nature of visual phenomenology is explained by the fact that we have perceptual access to a richly detailed world. Block (2007, 2011, 2014) argues that the richness of visual experience is explained by the fact that visual experience outstrips or “overflows” the limits of cognitive access. On Block’s view, phenomena such as change and inattentional blindness reflect limitations on visual cognition, not visual experience. Cohen *et al.* (2016) have sought to answer the puzzle by appealing to summary (or “ensemble”) representations that provide a singular description of a group. It goes without saying that none of these proposals are uncontroversial.]. In an elegant series of studies, Lau and colleagues have offered a novel answer to this long-standing question. They have proposed that our sense of the richness of visual phenomenology is an artifact of decisional and metacognitive biases. As they put it, phenomenology is “subjectively inflated.” As we will see, the group provides evidence that subjective inflation occurs in conditions of peripheral vision, visual crowding, and reduced attention [A thorough summary of the evidence in favor of subjective inflation is detailed by Knotts et al. (2018).]. Given the rigorous psychophysical techniques employed by these researchers, the proposal should be taken seriously as a potential solution to one of the most unrelenting puzzles in vision science.

The aim of this article is to critically evaluate this proposal. After some clarificatory remarks concerning the nature of subjective inflation, I turn to the representative findings invoked to support the “inflation” view put forward by Lau and colleagues. I argue that Lau and colleagues’ interpretation of these findings rests on questionable presuppositions concerning the relationship between decisional and metacognitive processes, on one hand, and visual phenomenology, on the other.

## Subjective Inflation

What exactly is “subjective inflation”? Lau and colleagues offer the following definition:

Inflation can be defined as the subjective overestimation of the reliability or quality of the sensory representations themselves … in inflation, the representations themselves are not necessarily filled in with details but are subjectively misestimated to be rich in content. Across the entire visual periphery, it is unlikely filling-in processes provide all the fine details in early sensory regions in a precise, pixelated representation instantly as soon as we view a scene (Odegaard *et al.* 2018, 2, citations omitted).

The latent assumption in this passage is that we seem to enjoy visual experiences that have precise, pixelated, and instantaneous richness. Lau and colleagues are taking this apparent snapshot-like richness of visual experience as their explanandum. According to Lau and colleagues, sensory representations or filling-in processes do not entirely account for this apparent richness (cf. Lau and Rosenthal 2011). Instead, this impression of snapshot-like richness must be rendered through an overestimation process that they refer to as “inflation.”

**Figure 1.**
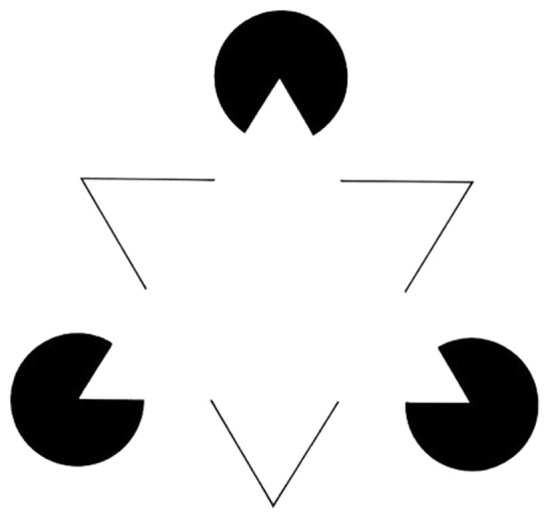
The Kanizsa Triangle. The triangle in the foreground is modally completed. The triangle in the background is amodally completed

**Figure 2.**
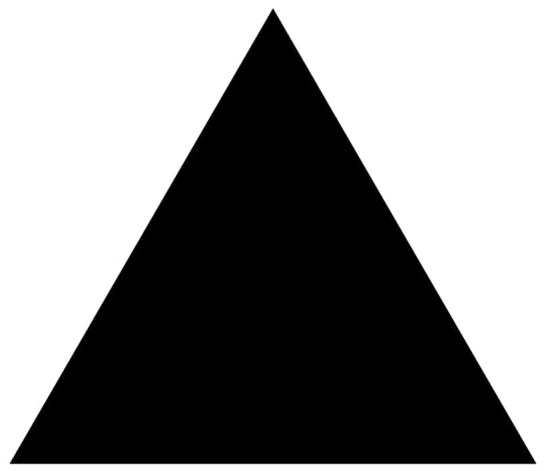
An ordinary triangle

How is this last step supposed to work? Lau and Brown (2019, 6–7) say that inflation increases the “strength” or “level” of phenomenology, making it “richer” and “more vivid.” It is plausible that one’s sense of subjective presence can vary in strength. The Kanizsa Triangle in Fig. 1 provides a nice illustration. It is easy enough to appreciate that the modally completed triangle in the foreground of the image has a stronger subjective presence than the amodally completed triangle in the background. One reason for this is that there is both boundary and featural completion in the case of the modally completed triangle, whereas this is lacking in the case of the amodally completed triangle (see Pessoa *et al.* 1998). Both the modally and amodally completed triangle have a weaker subjective presence than the non-completed triangle in Fig. 2. Perhaps we should think of subjective inflation as in some way strengthening subjective presence [This is not to say that the neural mechanisms underlying subjective inflation and perceptual completion will overlap (see Odegaard et al. 2018).].

It is worth flagging that a latent assumption underlying the present discussion—that visual experience seems to have a snapshot-like richness—is not universally accepted. Noë (2002, 2004) argues that such a characterization of visual experience subverts visual phenomenology. As he puts it, such a “… snapshot conception of visual experience… is not one to which perceivers themselves are committed. Perhaps it is an idea about the perception that psychologists or philosophers find natural. Perhaps it is way of describing experience that many ordinary perceivers would be inclined to assent to if they were asked appropriately leading questions. But this is compatible with its being the case that we do not really take our experience to be this way” (2002, 4–5). Although Lau and colleagues never explicitly endorse the snapshot conception, it is widely and implicitly presupposed (cf. Cohen *et al.* 2016), and the snapshot conception may be motivating the inflation view. In any case, I set this issue aside for the remainder of the discussion.

Talk of “subjective inflation” is ambiguous between two different interpretations. On a “visual inflation” interpretation, visual phenomenology is altered by decisional and metacognitive deficits. For instance, if visual inflation occurs, a liberal detection bias for a certain class of stimuli would generate an inflated visual sense of the presence of those stimuli. On a “cognitive inflation” interpretation, these deficits alter one’s cognitive (but not visual) phenomenology. Extending the previous example, if cognitive inflation occurs, a liberal detection bias for a certain class of stimuli would generate an inflated cognitive sense of the presence of those stimuli, perhaps through the formation of a conscious belief. These two options are not exhaustive. A further option would be to claim that these deficits have no phenomenological effects whatsoever; yet, this would not be a kind of subjective inflation.

Lau and colleagues clearly have visual inflation in mind when discussing subjective inflation. For instance, one study by the group is concerned with the possibility that “…liberal biases reflect inflated visual phenomenology” (Li *et al.* 2018, 1326). Another is entitled: “A Decisional Account of Subjective Inflation of Visual Perception at the Periphery”, where the focus is on “…subjective inflation at the perceptual level” (Solovey *et al.* 2015, 268). Similar remarks can be found in the other studies used to support the inflation view.

It is worth noting one additional ambiguity in the inflation view. The proposal leaves open two ways of interpreting the relation between visual phenomenology, on the one hand, and various decisional and metacognitive processes, on the other. On the “causal” interpretation, decisional and metacognitive processes cause visual phenomenology to be inflated. Accordingly, decisional and metacognitive processes enjoy a distinct existence from visual phenomenology; nevertheless, these processes systematically affect visual phenomenology, making visual inflation into a kind of cognitive penetration. On the “constitutive” interpretation, decisional and metacognitive processes partially constitute visual phenomenology. On this interpretation (but not on the causal interpretation), part of what it is to have inflated visual phenomenology is to have liberal decisional and/or metacognitive biases. It seems to me that Lau and colleagues have the constitutive interpretation in mind, and I will proceed on the basis of this assumption. That said, the issues I raise apply even if the causal interpretation is accepted.

## Evidence for the Inflation View

I now critically evaluate evidence in favor of the inflation view, suggesting that it is equivocal. If I am correct, we ought to adopt a position of agnosticism as to whether visual inflation occurs. To begin, consider a recent peripheral vision study by the group, in which subjects were required to perform a simple stimulus detection task (Solovey *et al.* 2015). The stimuli could be presented in either foveal or peripheral regions of each subject’s visual field. As expected, detection sensitivity was lower for stimuli presented in the periphery. To provide an adequate comparison, experimenters increased the contrast of the peripheral stimuli so that perceptual sensitivity was matched between foveal and peripheral stimulus presentations. The interesting finding was that, relative to foveal stimuli, subjects were more likely to indicate that they saw a peripheral stimulus in both stimulus-present and stimulus-absent trials (i.e. they exhibited a liberal detection bias for peripheral stimuli). This effect persisted even after giving trial-by-trial feedback, suggesting that it does not reflect a deliberate response strategy. This result has recently been extended to unattended, peripheral stimuli displayed in naturalistic settings (Li *et al.* 2018). Lau and colleagues claim that these results “…support the idea that in everyday visual experience, there is subjective inflation of experienced detail in the periphery, which may happen at the decisional level” (Li *et al.* 2018, 1325).

Suppose we take the main finding of this study—that subjects exhibit liberal detection biases for peripheral stimuli despite trial-by-trial feedback—at face value. It is often assumed that detection biases reflect post-perceptual processes and, as such, would not be taken as evidence in favor of visual inflation. Suppose for a moment that this was correct. Would the story be any different for liberal detection biases that are resistant to feedback? Lau and colleagues seem to think so, claiming that “these results may intrinsically reflect the perceptual experience of peripheral vision, because they were robustly replicated even under trial-by-trial feedback” (Solovey *et al.* 2015, 268; see also Knotts *et al.* 2018). It is true that many cognitive biases can be adjusted in light of new evidence. Yet, a range of biases that are clearly cognitive in nature, such as racist, religious, and superstitious biases, are also resistant to feedback, at least to a degree that they cannot be dismantled over the course of a few short hours. My deeply superstitious friend will knock on wood no matter how much evidence you provide in favor of the causal inefficacy of the act. Why think that liberal detection biases that are resistant to feedback are any different?

Lau and colleagues cite a paper by Witt *et al.* (2015) as evidence that “…detection biases could in principle reflect both subjective perception and decisional or response strategies…” (Li *et al.* 2018, 1325–1326). It is worth emphasizing that Witt and colleagues are making a largely technical point. They argue that sensitivity (*dʹ*) and criterion (*c* and *β*) measures from signal detection theory are terms of art that do not always neatly map onto perceptual and decisional processes, respectively:

While it is true that a change in decision processes will, all else being equal, affect a criterion measure (such as *c* or *β*), it is not true that a change in the criterion measure necessarily implies a change in decision processes. Similarly, while a change in *d′* can imply an effect on perception, it is not true that the absence of an effect on *d′* implies the absence of a perceptual effect. Even when *d′* remains constant, a perceptual effect can be quite large and show up as a large (and selective) change in the criterion measure across conditions (Witt *et al*. 2015, 289).

Yet, Lau and colleagues are not making an interpretive point about the criterion measure in signal detection theory. In fact, they appear to accept the orthodox construal of the criterion measure. They suggest that decisional and/or metacognitive processes underlie the liberal detection biases exhibited by subjects in their experiments (Li *et al.* 2018, 1331) and claim their study provides “…evidence that participants adopted more liberal criteria for making detection judgments when the target was unattended and presented in periphery” (Li *et al.* 2018, 1329). The tendentious claim underlying inflation views is that decisional and/or metacognitive processes can generate inflated visual phenomenology. The consideration raised by Witt and colleagues—that the criterion measure may in certain circumstances reflect a perceptual process—is orthogonal to this claim.

If Lau and colleagues were to claim that the liberal criteria effects were a result of a perceptual process, abandoning their claim that decisional and/or metacognitive processes underlie these criteria effects, it would be unclear how their position differed from a proposal according to which rich perceptual phenomenology is the result of the perceptual system “filling-in” details in unattended, crowded, and peripheral regions. However, Lau and colleagues are very clear that their proposal is supposed to be distinct from a filling-in proposal (Odegaard *et al.* 2018, 2).

Lau (personal correspondence) maintains that the decisional and/or metacognitive processes in question may nonetheless count as perceptual. On this line of thought, there is a decisional component to perception. This recommendation is in broad accordance with the constitutive interpretation of the inflation view. Such a rejoinder is difficult to dispute. At the same time, it is unclear whether this recommendation is at all substantive. If, on one hand, the point is that there is a liberal sense of the term ‘perception’ whose extension includes decisional and/or metacognitive processes, then the inflation view is merely advocating a prescriptive change in the terminology of psychology, not offering novel evidence aimed at explaining the apparent richness of visual phenomenology.

If, on the other hand, there is some additional argument or evidence to be given in favor of the claim that there is a decisional component to perception, then it ought to be spelled out explicitly by proponents of the inflation view. It is of no help to simply cite that there is no perception without decision as a kind of axiom. It is also of no help to appeal to the success of signal detection theory for support. Although the notion of a perceptual decision does play a central role in signal detection theory, the notion is ambiguous between a detection or discrimination process that is intraperceptual and one that is post-perceptual. On the former interpretation, a perceptual decision is a detection or discrimination decision literally made by the perceptual system. On the latter interpretation, a perceptual decision is a post-perceptual detection or discrimination decision formed on the basis of information provided by the perceptual system. Only the intraperceptual interpretation supports the idea that perception has a decisional component. Why should we favor the intraperceptual over the post-perceptual interpretation?

On that note, liberal detection biases are not the only form of evidence that have been used to support the inflation view. Consider a task by Odegaard *et al.* (2018) which required subjects to discriminate the orientation of visually crowded or uncrowded stimuli. Following each trial, subjects provided scaled ratings corresponding to how confident they were in their perceptual judgments. Although discrimination sensitivity was lower in crowded than in uncrowded conditions, confidence ratings were higher (More specifically, across trials in which subjects discriminated orientation incorrectly, confidence ratings were significantly higher in crowded than in uncrowded conditions. Across correct trials, confidence ratings were approximately equal.). Using a measure of metacognitive efficiency known as the “M-ratio,” experimenters showed that subjects’ confidence ratings did not effectively track their accuracy on the task, exhibiting a form perceptual decision-making that is suboptimal from a purely decision-theoretic point of view [Indeed, suboptimal perceptual decision making is quite common (for an extensive review, see Rahnev and Denison 2018).]. The Lau and colleagues take these results to show that “… far from perceiving [crowded stimuli in] the visual periphery with a high degree of fidelity, our subjective sense of the visual surround is inflated” (Odegaard *et al.* 2018, 7, citations omitted). The idea is that subjects’ erroneous overconfidence in crowded conditions—which drives the reduction in metacognitive efficiency—is evidence for visual inflation.

Again, however, it is unclear why confidence provides evidence in favor of visual inflation. Conceptually, confidence concerning one’s perceptual judgments appears doubly dissociable from visual phenomenology. Some subject *S* might be quite confident in their perceptual judgments concerning some stimulus *k* even when *k* is not visually experienced by *S.* For example, if *S* holds their eyes firmly shut, then *k* will not be visually experienced by *S* no matter how confident *S* is in their perceptual judgments concerning *k.* Furthermore, *S* may be entirely lacking in confidence concerning their perceptual judgments without any effect whatsoever on visual phenomenology. A student deep in the thick of Cartesian skepticism may lack confidence in all their perceptual judgments, but it would be preposterous to claim that reading Descartes might render one visually impaired.

Lau and colleagues may find these examples unconvincing, discounting them as limit cases. Nevertheless, the more pressing question remains: Why think that a high level of confidence implies anything about visual phenomenology? If Lau and colleagues are to avoid giving a question-begging reply (viz., a reply that presupposes their favored higher-order thought theory of consciousness), they must provide some mediating link between confidence and visual phenomenology. Without any such link, it is not obvious why the previous study gives us reason to accept the inflation view.

Lau and colleagues appear to be aware of the tenuous relation between confidence and visual phenomenology. Citing an earlier replication of results similar to those of Solovey *et al.* (2015), they suggest that “It is the joint observation, that peripheral perception leads to both erroneous overconfidence and liberal detection bias, that led us to think these findings may be relevant for subjective [visual] phenomenology” (Odegaard *et al.* 2018, 8; see also Lau and Brown 2019). Yet, as noted above, liberal detection biases also fail to provide univocal evidence in favor of visual inflation. Therefore, such an appeal is of no use.

Lau and colleagues also mention blindsight—a neurophysiological disorder resulting from damage or destruction of the primary visual cortex (i.e. V1) which appears to disrupt visual awareness while leaving certain perceptual capacities intact—as a case in which confidence provides a useful means of assessing whether visual awareness is present or absent (Odegaard *et al.* 2018, 8). In support of this claim, they cite an article by Ko and Lau (2012) which characterizes blindsight using a signal detection theoretic framework. On this framework, certain “essential” psychological properties of blindsight patients are explained by their poor metacognitive capacities. What are these “essential” psychological properties of blindsight? As Ko and Lau put it:

Although we are interested in the nature of the conscious experience in blindsight, this is hard to define clearly and is therefore less suitable to be the target of explanation for a formal computational theory. Fortunately, blindsight is a well-studied phenomenon. This means we can focus on certain patterns of behavior that presumably reflect the disturbed nature of conscious awareness (Ko and Lau 2012, 1402).

The “patterns of behavior” in question are none other than detection/discrimination criterion responses and confidence ratings. In other words, Ko and Lau assume, without any argument, that criterion responses and confidence ratings are an effective means of assessing the visual phenomenology of blindsight patients. In turn, Ko and Lau go on to provide an explanation as to why blindsight patients exhibit such patterns of behavior, an explanation that appeals to their impaired metacognitive capacities. What is important for our purposes is that, in the present dialectical context, invoking Ko and Lau’s signal detection theory framework to argue for a link between confidence and visual phenomenology is circular. The framework presupposes that confidence is an effective means of assessing visual phenomenology.

Notwithstanding, the case of blindsight might be taken to provide inductive support in favor of the existence of a link between confidence and visual consciousness if one antecedently assumes that visual awareness is impaired in blindsight patients. However, even if this is right, a lack of confidence and an absence of visual phenomenology might be coextensive in blindsight patients for reasons that are of no help to the inflation view. Ignoring any counterexamples that may arise, we might grant that, *ceteris paribus*, visual consciousness engenders confidence (The *ceteris paribus* clause is intended to exclude cases such as the Cartesian skepticism example given above. I take it that providing a more substantive, non-circular characterization of this clause would be quite challenging.). Ordinarily, when I enjoy a visual experience as of a scruffy dog in front of me, I become more confidence that there is a scruffy dog in front of me. It would follow that, *ceteris paribus*, my lack of confidence that there is a scruffy dog in front of me makes it doubtful that I enjoy a visual experience as of a scruffy dog.

These considerations are all compatible with a relatively uncontroversial, unidirectional link between confidence and visual consciousness: confidence often flows from visual consciousness. Such a unidirectional link captures the sentiment that:

…it is…unclear whether conscious experiences regarding a perceptual object can ever occur without any introspectable sense of certainty that something is presented to us. This is not at all to say that subjective confidence is equivalent to conscious perception, only that the latter entails at least some degree of the former (Odegaard *et al.* 2017, 9596, citations omitted).

Yet, from the simple observation that visual consciousness is often a source of confidence, one cannot justifiably infer changes in the strength or level of visual phenomenology from increases in confidence. After all, visual consciousness is just one of many different sources of confidence. One possible reason why subjects in the experiment might be overconfident in their ability to discriminate crowded stimuli is that they can often discriminate objects surrounded by other objects in non-laboratory settings: they simply need to move their eyes and fixate them. Another possible reason for subjects’ overconfidence in the experiment is that they may stubbornly, tacitly, and wrongly believe that peripheral vision tasks are easy. Such a belief might even be highly resistant to feedback, much like a superstitious belief. Again, the evidence is equivocal.

To my knowledge, the most direct evidence for visual inflation comes from one of the group’s studies concerning attention (Rahnev *et al.* 2011). This perceptual discrimination task required subjects to discern the tilt of gratings that either did or did not receive an attentional precue. Participants gave higher-visibility ratings to gratings that did not receive an attentional precue, relative to those gratings that did receive a precue, when discrimination sensitivity was matched between these two cuing conditions. (Discrimination sensitivity was matched by increasing the contrast of the uncued gratings.) Again, these effects were not eliminated by giving trial-by-trial feedback or by changing the task payoff. These results are taken to indicate that visual inflation occurs in the absence of focal attention (see also Lau and Rosenthal 2011).

Although visibility and confidence ratings diverge in a handful of detection and discrimination tasks [see, e.g. Sandberg *et al.* (2010) and Rausch and Zehetleitner (2016), respectively], these ratings usually correlate. In some circumstances, it is likely that visibility ratings are notational variants of confidence ratings. As Block (2019) puts it: “It is often said that when you give subjects a 4-point rating scale it doesn’t much matter whether you ask them to rate visibility or to rate confidence in their judgment. The pragmatic situation dominates the responses independently of exactly what the ratings are supposed to mean.” Thus, the use of visibility rather than confidence ratings in this study may be, at most, a superficial difference. One prediction of this claim is that similar results should obtain if the visibility ratings in this study were replaced by confidence ratings. A study by Rahnev *et al.* (2012) at least partially vindicates this prediction by showing that higher confidence ratings are given in a motion discrimination task when pre-stimulus neural activity is reduced in the dorsal attentional network, which likely reflects reduced task-related attention by the study’s participants.

In fact, Lau and colleagues might very well agree that visibility and confidence ratings are only nominally different. This is because they propose that both visibility and confidence ratings are, in essence, decision criteria effects (see Rahnev *et al.* 2011, supplement). As they put it, “the criteria for high visibility rating in discrimination should behave similarly to the criterion for detection” (Rahnev *et al.* 2011, 1514). That is, much like a detection criterion, visibility ratings reflect a process in which a response is made when an internal signal passes some decisional threshold. The upshot is that visibility ratings provide no additional support for inflation view unless one antecedently accepts a substantive assumption concerning the relation between decisional and/or metacognitive processes, and visual phenomenology, which is precisely what is under dispute (Of course, this study does provide additional data in support of the claim that some (subjective or non-subjective) form of inflation occurs).

To be clear, I am not suggesting that visibility ratings are useless to a science of consciousness. Rather, my suggestion is that we should question whether high visibility ratings are an indicator of apparent visibility if they are, at bottom, just decision criteria effects—as Lau and colleagues readily admit. This hesitancy to take visibility ratings at face value is not revolutionary: we would never take visibility ratings seriously if they were the result of experimenter bias, for instance.

## Conclusion

I conclude that, despite its ingenuity, the inflation view rests on evidence that is ripe for reinterpretation. All the results taken to support the inflation view are consistent with a view according to which decisional and metacognitive processes do not increase the strength of subjective presence in visual experience. It follows that, without additional evidence or argument, we ought to remain agnostic about the inflation view. This holds true despite the fact that the studies discussed use superficially different subjective measures (e.g. “yes-no” responses, confidence ratings, visibility ratings).

Much of the present discussion is an instance of a more general problem for the scientific study of consciousness: How do we effectively measure a subject’s conscious experience? As our discussion illustrates, this methodological question arises forcefully when subjective measures of consciousness, such as visibility ratings, conflict with objective measures, such as discrimination sensitivity. At this point, there is a lack of agreement as to how conflicts between different measures of consciousness ought to be resolved (see Seth *et al.* 2008; Sandberg *et al.* 2010; Irvine 2013).

There are other lingering questions that we have not addressed. Why do we exhibit liberal detection biases for peripheral stimuli? Why are we overconfident in our capacity to discriminate crowded and unattended stimuli? Notice that the inflation view does not itself provide an explanation of these findings (Inflation is, by definition, a process of overestimating our sensitivity to such stimuli, so it would be question begging—in the philosophers’ sense—to explain such findings by appealing to inflation, in much the same way that it would be question begging to attempt to explain opium’s tendency to cause people to sleep by appealing to its “dormitive virtue”). Rather, the inflation view (purports to) explain the apparent richness of perceptual experience, drawing on these findings for support.

One potential explanation, favored by several proponents of the inflation view, is that these findings stem from an inflexibility in perceptual decision-making. An issue with this proposal is that the results of the detection and discrimination tasks used in support of inflexible perceptual decision-making models [e.g. the “common criterion” models of Gorea and Sagi (2000), Rahnev *et al.* (2011), and Morales *et al.* (2015)] can be fit equally well to models in which perceptual decision-making is flexibly updated (see Denison *et al.* 2018, supplement). Indeed, the results of a more recent perceptual categorization task which avoids this underdetermination problem are at odds with at least some of the predictions of inflexible perceptual decision-making models (Denison *et al.* 2018). Although there is clearly more to say on this topic, if the present considerations are correct, it is questionable whether visual consciousness will play a central role.
